# Breast Cancer Incidence and Risk Reduction in the Hispanic Population

**DOI:** 10.7759/cureus.2235

**Published:** 2018-02-26

**Authors:** Eric J Power, Megan L Chin, Mohamed M Haq

**Affiliations:** 1 College of Medicine, Baylor College of Medicine

**Keywords:** breast cancer, hispanic, risk factors, risk assessment, cancer prevention

## Abstract

Breast cancer is the most common non-skin cancer amongst women worldwide and is the fifth leading cause of cancer-related mortality overall. It is also the foremost reason for cancer-related mortality in Hispanic females in the United States (US). Although the current incidence of breast cancer is significantly lower in Hispanics compared to that of non-Hispanic Whites (NHW) and Blacks, (91.9, 128.1, and 124.3 per 100,000, respectively, annually), this may increase if Hispanics develop similar lifestyle behaviors to other American women, in categories such as weight management, age at first birth, number of children, and breastfeeding habits. Stage-for-stage mortality for Hispanics is similar to NHWs, but the mortality rate is not declining as rapidly in this ethnic group. Hispanic women share many of the same risk factors for developing breast cancer as NHWs and Blacks. This suggests that many of the risk reduction strategies used in other racial populations may also benefit this group. Providing education about breast cancer and implementing risk reduction strategies in culturally-aware environments could help keep incidence low and reduce cancer-related mortality. Since Hispanics are the largest minority group in the US, this could have a significant impact on the incidence and mortality nationally.

## Introduction and background

Worldwide, breast cancer is the deadliest cancer amongst females in developing countries, causing about half a million total deaths each year [4]. Over a quarter of a million women were projected to develop breast cancer in the United States (US) in 2017, making it the most common non-skin cancer amongst American women [1]. The incidence of breast cancer in the US, as well as the age-standardized rate (ASR), are amongst the highest in the world [2]. An American female has about a one out of eight lifetime risk of developing breast cancer [3]. Breast cancer was predicted to cause about 40,000 deaths in women in the US in 2017, ranking second to lung cancer [1]. Morbidity and mortality from breast cancer will likely continue to rise as life expectancy increases globally.

Even within the same country, incidence and mortality vary significantly based on several risk factors, best organized as factors that increase the risk of developing breast cancer and factors that increase mortality after diagnosis [3]. Considerations which influence the risk of developing breast cancer include age, race, family history, genetics, lifestyle, and hormonal factors [3-7]. Other aspects, which have been shown to influence mortality after diagnosis, include socioeconomic status, education level, and access to care [8]. Although women in the Hispanic population share similar risk factors for developing breast cancer with non-Hispanic whites (NHWs) [5, 9-11], they have a lower incidence. However, they also have similar stage-wise survival rates and similar recent declines in mortality related to breast cancer [11]. The reason why Hispanics have a lower incidence is not fully understood but may be related to certain lifestyle factors, such as weight management, age at first birth, number of children, and length of breastfeeding [11]. These rates may change as more generations of Hispanics continue to undergo acculturation. This is supported by the fact that incidences among Hispanic ethnic subgroups in the US are higher than in their country of respective ethnic origin [2, 12].

Hispanics are the largest minority population in the US, and a comprehensive review of the incidence, mortality, risk factors, and risk reduction strategies related to breast cancer in the Hispanic population has not been conducted recently. In order to learn more about these topics, as well as explore possible mechanisms related to why Hispanics have lower incidence compared to other races, a search of the literature was conducted on PubMed. Keywords used in the search included but were not limited to Hispanic, breast cancer, cancer, mortality, incidence, risk factors, risk reduction, and genetics. Relevant articles published within the last 10 years were included in the review. Resources outside of these search parameters were included for information about the demographics and relevant statistics related to the Hispanic population in the US, as well as statistics about breast cancer in the general population.

## Review

The criteria for individuals considered to be a part of the Hispanic population has varied significantly over time. Some determinants used in the past included use of Spanish language in the home, surname, and birthplace of the individual or their parents [13]. However, since 1970, the racial classification for Hispanics was changed to include any person who identifies as "Hispanic" or "Latino" on the census questionnaire. The specific categories within Hispanic and Latino classification include Cuban, Mexican, Puerto Rican, South or Central American, or other Spanish culture or origin regardless of race [14]. Although self-identification is now used for census data collection, the methods used for racial classification may be different in scientific research [15]. The Hispanic population in the United States exceeded 57 million people in 2015, about 18% of the total U.S. population. This population is one of the fastest growing ethnic groups in the country, totaling over half of the population growth between 2000 and 2014, and is estimated to represent 25% of the overall population by 2050 [16-17]. The majority of Hispanics in the US are Mexican-American, representing 64% of the total Hispanic population. The second and third largest subgroups of Hispanics are those of Puerto Rican and Cuban descent, comprising 9.5% and 3.7% of the Hispanic population, respectively [18].

Incidence and mortality

Overall Incidence of Breast Cancer in the US

According to the North American Association of Central Cancer Registries (NAACCR), between 2009 and 2013, the overall incidence of breast cancer in the US was 123.3 cases per 100,000 annually [19]. The US has the eighth highest ASR of any country worldwide [2]. Breast cancer incidences within the US vary significantly based on factors such as socioeconomic status, geographic location, race, and ethnicity. Incidence rates have been positively correlated with socioeconomic status throughout life [20]. Females living in neighborhoods with a higher average income have as much as a two-fold increase in incidence [21]. Breast cancer incidence is significantly different in various regions of the US. The Northeast states, for the most part, have higher incidences than the national average, while states in the Southwest have lower incidence rates. New Hampshire has the highest incidence of any state at 144.9 cases per 100,000, while Nevada has the lowest incidence at 106.6 [22]. Breast cancer incidence also differs based on race and ethnicity. NHWs have the highest rate (128.1), followed by Blacks, American Indian/Alaskan Natives, Hispanics, and Asian/Pacific Islanders with the lowest incidence (88.3 per 100,000) [8]. Overall, breast cancer incidence has remained stable amongst US women between 2003-2012 [23] after a slight decline from 2000-2003, possibly related to the decreased use of hormone replacement therapy (HRT) during that time.

Breast Cancer in Hispanics in the US

Hispanic women have about a 20% lower incidence of breast cancer than the general population of the US [3, 11]. Incidence in the Hispanic population was 91.9 per 100,000 annually between 2008-2012, compared to 128.1 and 124.3 for NHWs and African Americans, respectively [8]. Over the course of her lifetime, a Hispanic woman living in the United States has about a 9.8%, or one-in-10 chance of developing breast cancer. This is significantly lower than the national rate of 12.3%, or about one-in-eight [11]. Two studies conducted between 2003-2012 and 2006-2010 showed cancer incidence rates were stagnant or slightly declining in Hispanic women, with an average annual percentage decrease of 0.1% and 0.6%, respectively [3, 11]. Despite the low incidence compared to the national average, breast cancer is still the most common cancer among Hispanic women, accounting for an estimated 19,800 new cases in 2015, and representing 29% of all cancers diagnosed in this group [11].

Breast Cancer Incidence in Hispanic Ethnic Subgroups and Countries from Where US Hispanics Hail

**Figure 1:**
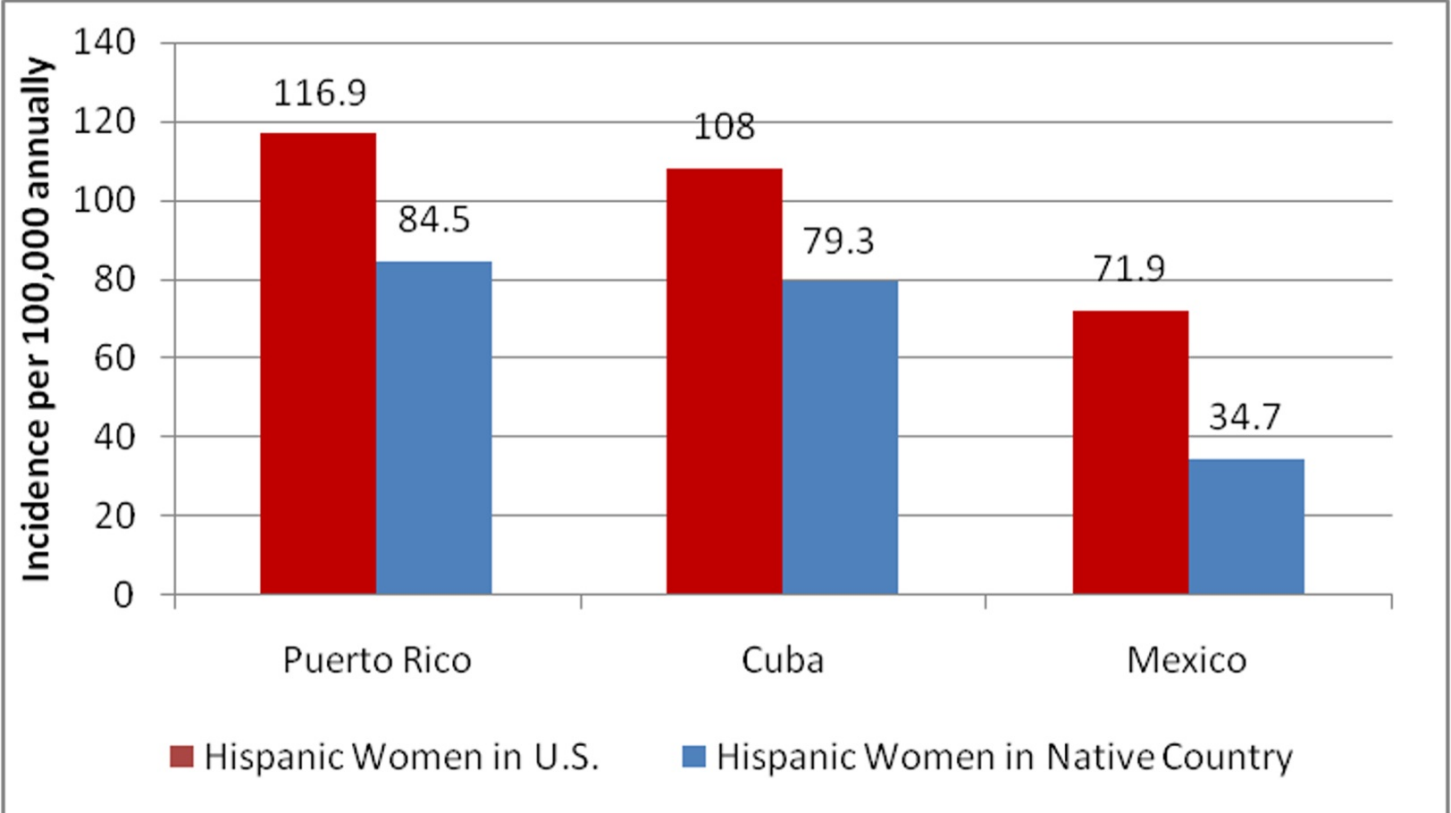
Incidence of breast cancer of Hispanic women in U.S. compared to countries of their ethnic origin Data from the World Health Organization Global Cancer Observatory (WHO GLOBOCAN) database in 2012, compared to data from a study in 2015 which recorded cancer rates amongst Hispanic subgroups in the U.S. reveals Hispanic women within the U.S. have a greater incidence of breast cancer than women in their countries of ethnic origin [2, 12]. U.S.: United States

Even within the Hispanic population, cancer incidence and mortality have been shown to vary significantly based on which country the women hail from [11, 12, 24]. A study conducted in Florida in 2001 suggested females of Puerto Rican and Cuban ethnicities had incidences of breast cancer more similar to NHWs than to Mexican-American Hispanics, with incidences of 116.9 and 108.0 per 100,000, respectively. Women of Mexican descent had the lowest incidence at 71.9 per 100,000 [12]. When compared to the World Health Organization Global Cancer Observatory (WHO GLOBOCAN) database from 2012, Hispanic women in the US had higher incidence rates than Hispanic women who lived in the country of their ethnic heritage. This difference was especially large in the Mexican-American population whose breast cancer incidence was more than twice as high as the Mexican population (71.4 vs. 34.7, respectively) (Figure 1). The relative incidence between the ethnic subgroups was the same among females in their native country and in the United States. The incidence of breast cancer among women in Puerto Rico was 84.5, while Cuba was 79.3, and Mexico was 34.7 per 100,000 annually (Figure 1) [2].

Mortality

The leading cause of death among all Hispanics in the United States is cancer. Amongst the types of cancer, breast cancer causes the most mortalities among Hispanic women, resulting in an estimated 2,800 deaths in 2015. This number represented 16% of all cancer-related deaths in this population in 2015 [11].

Even though breast cancer is the deadliest cancer among Hispanic women, they still exhibit 25-30% lower overall mortality rates compared to the US population [24]. Much of the lower mortality rates can be attributed to the lower incidence in this population. Breast cancer mortality has been decreasing for many races, and overall amongst females in the US. The Hispanic population has had a decline in mortality of 1.3% annually from 2003-2012, which is slightly slower than the 1.9% seen in NHWs during this time period [11]. Even though Hispanics are diagnosed in the localized stage of breast cancer slightly less often than NHWs (57% compared to 65%), Hispanics have a similar stage-for-stage mortality rate. This may be explained by the fact that more Hispanics are diagnosed in the regional stage (36% vs. 28%, respectively), which is still often associated with good clinical outcomes [11].

Mortality is proportional to incidence within Hispanic ethnic subgroups, with US Hispanics of Cuban ethnicity having the highest mortality rate at 18.9, followed by Puerto Rican at 17.0, and Mexican at 15.0 per 100,000 annually [11].

Clinical and biological factors

**Figure 2:**
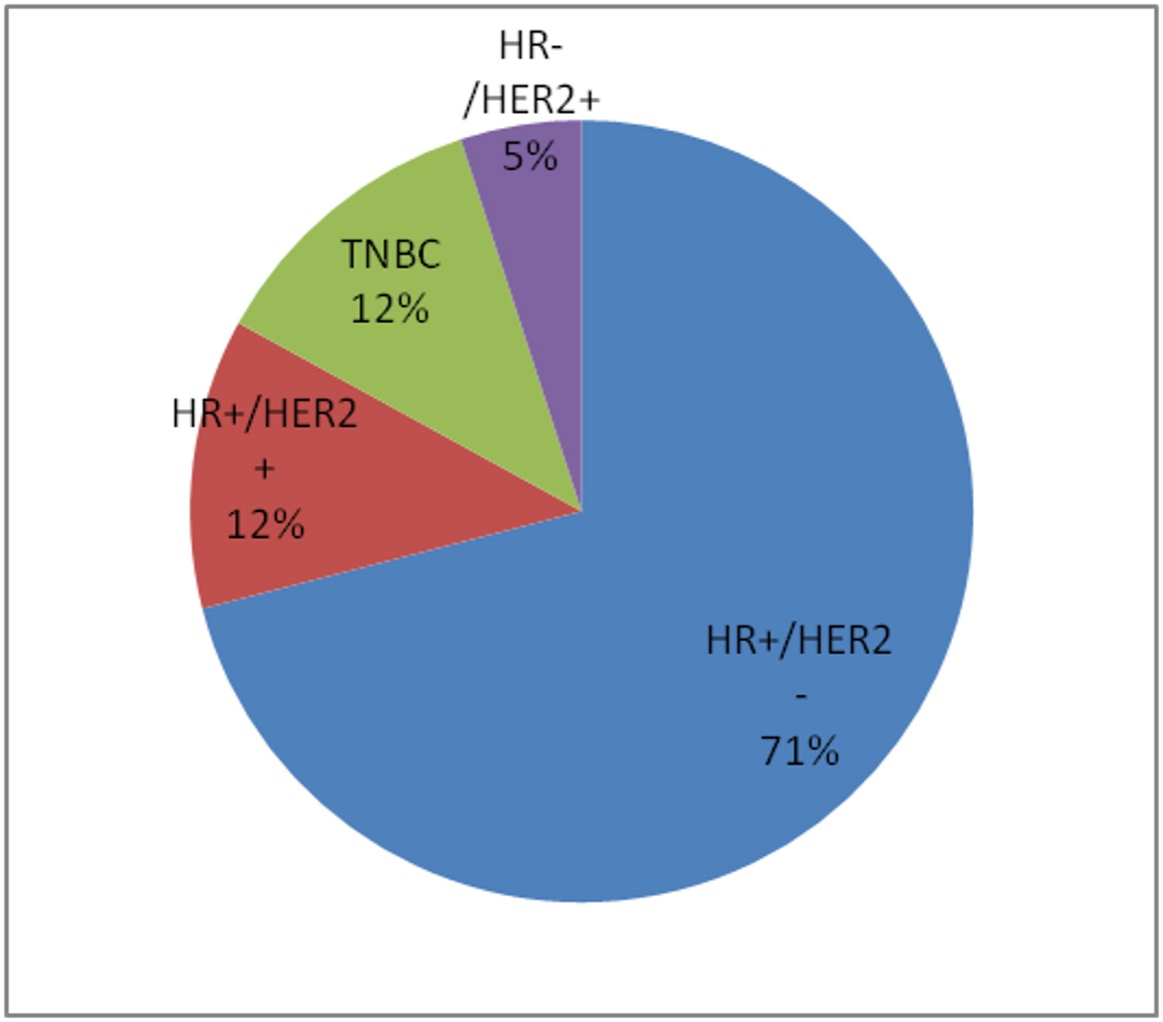
Prevalence of subtypes of breast cancers in U.S. Hispanic population The Hispanic population within the United States (U.S.) is predominantly HR+/HER2- [8]. HR: hormone receptor; HER2: human epidermal growth factor receptor-2 protein; TNBC: triple-negative breast cancer (HR-/HER2-)

Although the term "breast cancer" is commonly used to refer to a single disease, breast cancer has many distinct molecular and histologic subtypes [25]. Incidence rates of these various subtypes differ between races. As various subtypes of breast cancer are associated with different prognoses, it is important to look at incidence rates for the subtypes of breast cancer, along with incidence of breast cancer overall. Commonly assessed biological markers used to evaluate the subtype of breast cancer include hormone receptor (HR), also referred to as estrogen receptor/progesterone receptor (ER/PR), and human epidermal growth factor receptor-2 (HER2) protein [26]. The most common subtype among Hispanics is HR+/HER2- (luminal A). This represented 71% of all breast cancers diagnosed in Hispanics, slightly lower than NHWs (76%) and higher than African-Americans (62%). Luminal A breast cancer is associated with higher survival rates, partially due to increased responsiveness to hormonal therapy. Hispanics had similar rates of HR+/HER2+, HR+/HER2-, and HR-/HER2+ breast cancers as NHWs, but had significantly lower rates of HR-/HER2- or triple-negative breast cancer (TNBC) than African Americans (12% vs. 22%) [8]. The relatively high incidence of HR+/HER2- and low incidence of TNBC in the Hispanic population, as compared to African-Americans, likely contributes to lower mortality for Hispanics [24]. Figure 2 shows the incidence of each histologic subtype of breast cancer in the US Hispanic population.

Two genetic markers, not often used to describe a type of breast cancer, but rather to estimate an individual's predisposition to developing it, are the breast cancer 1 and breast cancer 2 (BRCA1 and BRCA2) genes. Mutations in these genes have been shown to significantly increase the risk of developing breast cancer as well as several other types of neoplasias. Screening to identify BRCA1 and BRCA2 carriers is especially important because there are several preventative options which can lower the risk of developing breast cancer. Analysis from a sample of 64,717 females who underwent genetic screening from 2006-2008 revealed the mutation rate of BRCA1 and BRCA2 was about the same in Hispanics and NHWs [5]. However, both Hispanic and African American women were 3.9 - 4.8 times less likely to undergo genetic testing than NHWs, resulting in a larger group of undiagnosed carriers of the BRCA1 and BRCA2 mutation in these populations. Hispanic and African American women were also found to be two and 16 times less likely, respectively, to discuss genetic testing for BRCA1 and BRCA2 with a clinical provider compared to NHWs [27]. A possible explanation for this discrepancy in testing includes lower average income and insurance levels, as well as less education about the benefits of genetic testing in the Hispanic and African American population.

Risk factors for breast cancer

There are several lifestyle and non-lifestyle factors which influence the likelihood of developing breast cancer. Some of these risk factors include menstrual history, reproductive factors, hormone use, as well as genetics, family history, and diet and exercise [6]. Hispanic women display differences in several of these risk factors compared to NHWs [28], which may contribute to their current lower levels of breast cancer incidence. However, Hispanics also face disparities in screening and genetic testing related to breast cancer compared to NHW women [29]. The Hispanic population overall is also more susceptible to having difficulties related to access to care, insurance coverage, and a higher prevalence of unmet healthcare needs [9].

Reproduction and Hormonal Factors

It is well known that several reproductive and hormonal factors influence the likelihood of developing breast cancer. Several factors which decrease risk include early age at first full-term birth, multiparity (having multiple children), and longer periods of breastfeeding [30]. Hispanics, on average, are younger during their first-pregnancies, have more children [28], and breastfeed slightly more often than NHWs at birth, six months, and 12 months [31].

Genetic Mutations

An important advancement in the prevention and treatment of breast cancer which has emerged is the availability of genetic testing. Testing for specific genetic markers, such as BRCA, in combination with family history, has allowed physicians to better estimate a woman's risk of developing breast cancer. One of the most frequently discussed mutations in breast cancer literature is the germline mutation in BRCA1 and BRCA2 genes, which accounts for about 25% of inherited breast cancers and 10% of all breast cancers. Although mutation frequencies have been shown to be similar between Hispanics and NHWs, it is estimated that Hispanics are four to five times less likely to receive BRCA screening [5].

Family History

If an individual has a family history of breast cancer or has inherited genetic mutations, such as BRCA1 and BRCA2, she is at a significantly higher risk of developing breast cancer. A woman with a single first-degree relative who has developed breast cancer has a one-and-a-half to two times greater risk of developing breast cancer herself. Having two first-degree relatives who have developed breast cancer raises an individual's risk about six-fold [32].           

Risk assessment for Hispanic women

In order to appropriately target risk reduction strategies to those who are most likely to benefit, it is necessary to estimate individual risk first. Current risk-assessment models incorporate risk factors in order to evaluate the proportional risk of developing breast cancer. One such model, the Gail model, uses several risk factors, such as current age, race, age at menarche, age at first live birth, first degree relatives with breast cancer, previous breast biopsies, and atypical hyperplasia on any previous breast biopsy to compare an individual’s risk to that of the average American [6, 33-34]. According to the American Society of Clinical Oncology (ASCO) and the National Comprehensive Cancer Network (NCCN), the threshold values for determining women at high-risk for breast cancer is at a 1.67-fold higher five-year cumulative risk of invasive breast cancer [6, 33]. Despite the wide use of the Gail model in assessing the risk of developing breast cancer, this model was created from data of only NHW women [34]. Currently, there is not a standard model used for assessing risk in US Hispanic women.

Efforts have been made to form a Hispanic-specific model through analysis of the established Breast Cancer Risk Assessment Tool (BCRAT) and newly explored models with collected data. Evaluation of the BCRAT, via the Gail model, of US Hispanic women revealed weaknesses of the tool. It does not address specific risk factors that could be attributed to Hispanics, such as migration history and country of origin [10]. This tool also requires calibration when assessing different populations at different times, since calibration has been shown to improve the accuracy of the predictive model. Even so, there is still consistent underestimation of the number of women at risk of invasive breast cancer [10]. In addition, the Gail model has not been tested in non-US populations and does not assign any significant value to a previous family history of breast cancer. Given that the Hispanic population in the United States is a diverse group made up of people and immigrants from multiple countries, these previously established models may not be best for analyzing risk in this group.

Beyond the efforts to calibrate the currently pre-existing BCRAT, data from the San Francisco Bay Area Breast Cancer Study (SFBCS), California Cancer Registry (CCR), and Surveillance, Epidemiology, and End Results (SEER) Program was used to develop the Hispanic Risk Model (HRM), the first nativity-specific risk model for Hispanic women [35]. The HRM allowed for absolute risk estimates of invasive breast cancer to be differentiated between US-born and foreign-born Hispanic women. Due to the nativity-specific focus of this model, some risk factors were omitted from the original Gail model and the nativity-specific relative risks from the SFBCS were incorporated. The HRM is a tool that needs to be developed further, as the development of it would be most suited for women with backgrounds similar to the SFBCS and CCR and requires inclusion of additional risk factors for increased accuracy [35]. It is, however, a positive first step in assessing risk of invasive breast cancer in the the Hispanic population within the US.   

Strategies for risk reduction in the Hispanic female population

The long latency from the start of carcinogenesis to the clinical presentation of breast cancer allows for multiple interventions. General risk reduction strategies, such as weight maintenance via diet and exercise, screening, and breast-feeding, are applicable to all women and could reduce the risk by as much as 40% [33]. Primary prevention strategies like these have a large effect on high-risk women and are often underutilized [36]. Additionally, there are risk reduction strategies for high-risk populations, such as those with family history of breast cancer, including genetic screening, medications, and surgical procedures [5-6, 33]. Optimally, genetic counseling and testing for BRCA1 and BRCA2 would be offered for women at high-risk in addition to regular screening to aid in the early detection of cancer. However, socioeconomic and lifestyle factors have been found to influence morbidity and mortality of minority races and ethnicities. Multiple explanations for socioeconomic and lifestyle disparities, such as insurance status, obesity rates, and screening availability, have been proposed over time. Specific strategies can be used to selectively target these barriers to treatment [37-38].           

Diet

Direct correlation between diet and breast cancer has been difficult to prove. Prospective studies do not support dietary fat as an etiology of breast cancer. Healthy dietary patterns are notably more important for reducing the risk of other morbidities, such as cardiovascular disease and diabetes, independent of weight and other lifestyle risk factors [39]. However, weight gain in adulthood is associated with an increased risk of breast cancer. While diet is not directly correlated with an increased risk of breast cancer, its effect on weight and total health should not be ignored. All patients, especially those at high risk of developing breast cancer should be advised to maintain a healthy diet for weight control [7].

Alcohol consumption, even in moderate amounts, has been shown to increase the risk of breast cancer [40]. Physicians should advise avoidance of alcohol in high-risk patients.

Exercise

Several organizations have recommended guidelines regarding diet and physical activity for prevention of several types of cancer, including the World Cancer Research Fund (WCRF) and the American Cancer Society (ACS). These guidelines suggest participating in at least 150 minutes of moderate intensity exercise weekly, maintaining a healthy weight (as lean as possible without being underweight), and eating a plant-based diet [7, 39]. Studies have suggested adhering to these guidelines could prevent up to 25 - 30% of all breast cancer cases [39]. An observational study suggested that Hispanics who follow the ACS lifestyle guidelines may see an even greater risk reduction in developing breast cancer than NHWs [7]. Table 1 illustrates possible strategies to change the lifestyle of patients and reduce their risk of developing breast cancer.

**Table 1:** General Risk Reduction Strategies The slow evolution of breast cancer allows for multiple types of lifestyle interventions to reduce risk [6, 33]. HRT: hormone replacement therapy

Types of Intervention	Mechanism of Action	Specific Approach	Relative Risk Reduction	
Childbearing patterns (first live birth under 18 vs. over 30 or null parity)	Early pregnancy leads to earlier terminal differentiation of ductal epithelium	Pregnancy; prenatal education	40%	
Breastfeeding	Causes maturation of ductal epithelium	Nursing education; providing spaces for nursing mothers at work and/or in public	4% reduction for every year of breastfeeding	
Avoidance of hormone supplements (HRT)	Unknown; HRT use is associated with breast cancer	Physician education	26%	
Post-menopausal weight management	Reduction in adipose tissue that stores hormones	Community and physician education; improved access to fitness facilities, training, and/or equipment	20-30%	
Healthy dietary habits and physical activity	Lowers body fat, enhances immune function, affects hormone levels, and delays menarche in children	Community education and legislation for mandated food labeling; Exercise education and planned/mandatory exercise programs	20-30%	

Additional Strategies to Improve Accessibility and Education

Breast cancer is more likely to be diagnosed at a later stage in Hispanic women than NHW despite having a lower incidence rate than NHW and Black women [38, 41-43]. Even after accounting for age, socioeconomic status, and methods of detection, these women are more likely to be diagnosed at a later stage and present with larger tumor size than NHW [38]. The disparity between ethnic minorities and NHW has been largely attributed to a lower frequency of mammograms, longer time between mammograms, and decreased timely follow-up of suspicious mammograms [43]. In addition, a lack of knowledge and information on cancer and culturally-based perceptions of cancer contribute to this difference.

Early detection awareness and education with culturally appropriate strategies are goals of programs meant to target and improve outcomes for Hispanic women. One program from the Moffit Cancer Center, the Yo me cuido (YMC) program, has been designed to reduce the disparities found among Hispanic women in Florida while also engaging the community [43]. YMC is an innovative program that has incorporated client reminders, media campaigns in Spanish, education materials in both English and Spanish, group and individual workshops, and funding assistance to increase awareness, education, and access to screening [43]. There are basic tenets that programs working with Hispanic communities have undertaken in recognition of the impacts of cultural values like respeto, personalismo, confianza, simpatía, and familia [41, 43]. Acceptance and understanding of these cultural characteristics are vital for programs with the goal of preventing breast cancer and improving outcomes for Hispanic women.

Breast Self-examination

Breast self-examination (BSE), despite its relatively simple use as a tool, has not been consistently shown to reduce breast cancer mortality. However, BSE does correlate with detection at an earlier clinical stage [33]. As such, BSE could be a valuable tool in combating breast cancer in populations where additional screening methods are not easily accessible. Hispanic women with breast cancer are more likely to be diagnosed with an advanced stage of breast cancer than NHW [38, 44]. Increased education and awareness of BSE could possibly assist in improving breast cancer prognosis in Hispanic women and similarly disadvantaged communities.

Screening

Annual or biennial mammography has been previously shown via randomized trials to be effective in reducing mortality in women aged 50 to 69 years [6, 33]. Data have not consistently shown a similar reduction in mortality among women aged younger than 50 years. Studies with more women screened could provide clearer insight on the preventative benefits of screening in women younger than 50 years in the future. Other studies have found that clinical breast exams should regularly accompany mammographic screening since about 15% of cancers missed by mammography can be detected this way [6, 33]. The combination of both types of exams will optimize early detection of breast cancer in women.

Medications

Selective estrogen receptor modulators (SERMs), like Tamoxifen and Raloxifene, have been shown to be effective for breast cancer prevention in large randomized trials [6, 33, 45-46]. This study included women aged 35 years or older with an assessed Gail model risk of over 1.67. Data aggregated from four separate studies illustrated a risk reduction of 38%. As such, it is currently recommended for women aged 35 years or older with a high risk of breast cancer, according to the Gail model, to take Tamoxifen for five years. Raloxifene has been proven via a randomized trial to be as equally effective as Tamoxifen in risk reduction [6, 33, 46]. As a second generation SERM, Raloxifene has the additional benefit of a lowered incidence of side effects, such as edema, hot flashes, and arthralgias, and thus may be more desirable than Tamoxifen.

Bilateral Total Salpingo-oophorectomy

Women with BRCA1 and BRCA2 mutations who receive bilateral total salpingo-oophorectomy at or before the age of 40 benefit from a 40% reduction in the risk of breast cancer [6, 33, 47]. These mutations also increase the risk of ovarian cancer, albeit at a much lower rate than that of breast cancer. A salpingo-oophorectomy thus decreases the risk of both ovarian and breast cancer.

Bilateral Total Mastectomy

According to two small prospective studies with relatively short follow-up, women with BRCA1 or BRCA2 mutations may benefit from a significant reduction in breast cancer risk following bilateral total mastectomy. Other studies with retrospective follow-up of 13 to 14 years revealed a 90% reduction of breast cancer risk in women with BRCA1 and BRCA2 mutations who underwent bilateral total mastectomy [6, 33, 48].

## Conclusions

The significant growth of the Hispanic population is expected to continue over the next few decades, which will likely result in an increase in the overall number of Hispanic females in the US with breast cancer. Continued acculturation of this population and decreased access to care are factors which could result in a rise in incidence and mortality related to breast cancer in Hispanic women. The slow clinical evolution of breast cancer and the presence of many modifiable risk factors make it an ideal disease for preventive interventions. Resources are more effectively utilized in prevention strategies than in treating advanced cancers. The risk of acquiring breast cancer can be reduced by modifications in diet, exercise, weight management, and preventive health screening. More programs dedicated to the health of Hispanic women should be utilized in order to increase education and preventative screening among Hispanic women. This will require a joint effort between physicians, local communities, research, government, and nonprofit institutions.
